# Functional significance of Blood Oxygen Level Dependent (BOLD) imaging in patients with coronary artery disease - a validation study using fractional flow reserve

**DOI:** 10.1186/1532-429X-13-S1-P92

**Published:** 2011-02-02

**Authors:** Judy Luu, Jodi Harker, Dominik Guensch, James Hare, Matthias Friedrich

**Affiliations:** 1 University of Calgary, Calgary, AB, Canada; 2 Baker IDI Heart & Diabetes Institute, Melbourne, Australia

## Background/purpose

Blood oxygen level-dependent (BOLD) cardiac MRI (CMR) uses the signal generated by endogenous hemoglobin in the blood supply to directly measure tissue oxygenation. Therefore it may be useful as a non-contrast enhanced, non-invasive method to detect the presence of myocardial ischemia in patients suspected of having coronary artery disease (CAD). The aim of this study was to validate whether BOLD-sensitive CMR images can detect and quantify alterations in myocardial oxygen levels in patients with CAD, in comparison to the gold standard of fractional flow reserve (FFR).

## Methods

Oxygen-sensitive BOLD CMR scans were performed in patients who were scheduled for clinically-indicated coronary angiography. BOLD images were captured during rest and adenosine-induced coronary hyperemia. The mean BOLD signal intensity (SI) percent changes were calculated between rest and hyperemia in the subendocardial myocardium at basal, mid, and apical regions. Segmental ΔSI% in the corresponding coronary territory was defined as ischemic (using a cut-off of <0.80) or non-ischemic by FFR. The BOLD segment with the lowest ΔSI% in the territory subtended by the FFR measurements was selected for statistical analysis. Bland-Altman analysis was used to assess the level of agreement between all segments analyzed by two blinded readers.

## Results

**Figure 1:**
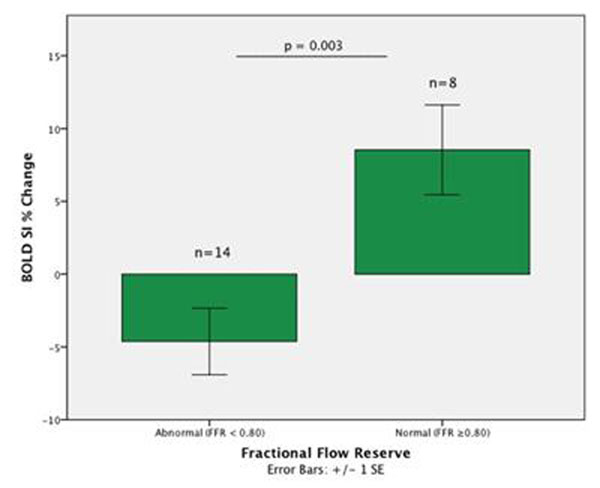
Comparison of BOLD Signal Intensity Percent Changes to FFR Pressure Wire Measurements

**Figure 2:**
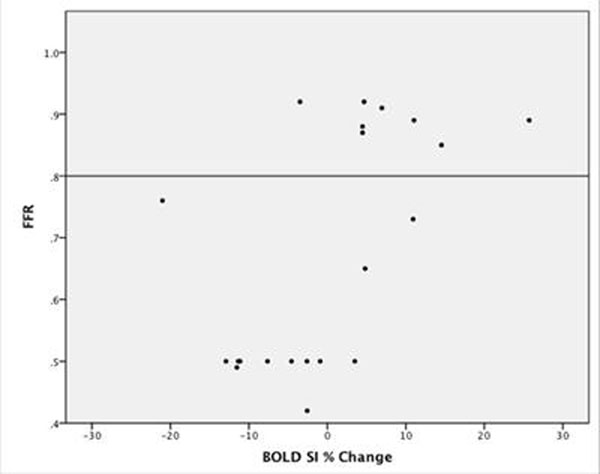
Comparison of FFR Pressure Wire Measurements as a Continuous Variable to BOLD Signal Intensity Percent Changes

**Figure 3:**
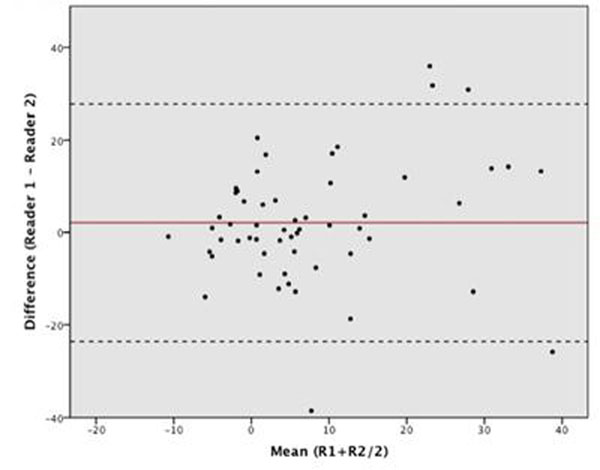
Bland-Altman analysis for inter-observer variability

Twenty-eight patients totaling 147 myocardial segments were available for analysis. 73 segments were excluded, with 66% of these being apical. The remaining 74 segments equated to 22 patients (60 +/- 9y, 19 males), eight of these had a normal FFR (≥ 0.80) and 14 had FFR values <0.80. Mean BOLD SI percent change was significantly less in patients with abnormal FFR values (-4.62 +/- 2.28% SEM), in comparison to patients with normal FFR values (8.54 +/- 3.08 % SEM); p=0.003. (See Figure 1 and 2). The Bland-Altman analysis indicated that the 95% limits of agreement between the two readers ranged from -23.6% to 27.8% (Figure 3).

## Conclusion

Our pilot data indicate that BOLD-sensitive CMR may allow for selectively identifying hemodynamically relevant coronary artery stenoses based on a blunted hyperemic response to adenosine. Image quality (particularly in apical segments) remains a significant limitation of the BOLD technique, with this reflected in suboptimal interobserver variability. Most excluded segments were from early studies, suggesting improved acquisition quality with experience. Further recruitment will enable validation of sensitivity and specificity with identification of a standard threshold for ischemia identification.

